# Cisplatin‐induced oxPAPC release enhances MDSCs infiltration into LL2 tumour tissues through MCP‐1/CCL2 and LTB4/LTB4R pathways

**DOI:** 10.1111/cpr.13570

**Published:** 2023-10-31

**Authors:** Ji Nie, Jiayuan Ai, Weiqi Hong, Ziyi Bai, Binhan Wang, Jingyun Yang, Ziqi Zhang, Fei Mo, Jing Yang, Qiu Sun, Xiawei Wei

**Affiliations:** ^1^ Department of Biotherapy, Laboratory of Aging Research and Cancer Drug Target, State Key Laboratory of Biotherapy, National Clinical Research Center for Geriatrics West China Hospital, Sichuan University Chengdu Sichuan China; ^2^ Department of Pulmonary and Critical Care Medicine, The First People's Hospital of Yunnan Province The Affiliated Hospital of Kunming University of Science and Technology Kunming Yunnan China; ^3^ West China Medical Publishers, West China Hospital, Sichuan University Chengdu Sichuan China

## Abstract

Lung cancer is the leading global cause of cancer‐related death, however, resistance to chemotherapy drugs remains a huge barrier to effective treatment. The elevated recruitment of myeloid derived suppressor cells (MDSCs) to tumour after chemotherapy has been linked to resistance of chemotherapy drugs. Nevertheless, the specific mechanism remains unclear. oxPAPC is a bioactive principal component of minimally modified low‐density lipoproteins and regulates inflammatory response. In this work, we found that cisplatin, oxaliplatin and ADM all increased oxPAPC release in tumour. Treating macrophages with oxPAPC in vitro stimulated the secretion of MCP‐1 and LTB4, which strongly induced monocytes and neutrophils chemotaxis, respectively. Injection of oxPAPC in vivo significantly upregulated the percentage of MDSCs in tumour microenvironment (TME) of wild‐type LL2 tumour‐bearing mice, but not CCL2−/− mice and LTB4R−/− mice. Critically, oxPAPC acted as a pro‐tumor factor in LL2 tumour model. Indeed, cisplatin increased oxPAPC level in tumour tissues of WT mice, CCL2−/− and LTB4R−/− mice, but caused increased infiltration of Ly6C^high^ monocytes and neutrophils only in WT LL2‐bearing mice. Collectively, our work demonstrates cisplatin treatment induces an overproduction of oxPAPC and thus recruits MDSCs infiltration to promote the tumour growth through the MCP‐1/CCL2 and LTB4/LTB4R pathways, which may restrict the effect of multiple chemotherapy. This provides evidence for a potential strategy to enhance the efficacy of multiple chemotherapeutic drugs in the treatment of lung cancer by targeting oxPAPC.

## Graphical abstract

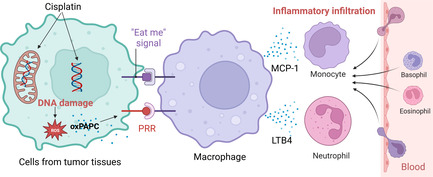

## 1 INTRODUCTION

Lung cancer (LC) is the main cause of malignancy‐related mortality globally because of its late diagnosis and poor results.^1^, ^2^ It is split into two types: small cell lung cancer and non‐small cell lung cancer, with non‐small cell lung cancer accounting for around 85% of all cases.^3^ Until now, platinum‐based chemotherapy has been the first‐line chemotherapy regimen for the treatment of LC, particularly for non‐small cell lung cancer.^4^ The formation of platinum‐DNA adducts to induce DNA damage and thus tumour cells apoptosis is the primary mechanism of their anti‐tumour activity.^5^, ^6^ While early treatment has proven to be more effective, the complete eradication of cancer cells remains a significant obstacle for most existing anti‐tumour agents. The vast majority of patients experience a decline in treatment efficiency or tumour recurrence in the later stages of cisplatin (DDP) therapy, and tumours may develop intricate chemotherapy resistance mechanisms in vivo, depending largely on their interaction with host factors.^7^

TME is a highly intricate and continuously evolving system that encompasses several types of stromal cells, including fibroblasts and endothelial cells, and various innate and adaptive immune cells.^8^ It has emerged as a crucial factor in cancer growth and metastasis, impacting patient outcomes.^9^, ^10^ MDSCs consist of highly heterogeneous cells that originate from immature myeloid progenitor cells. They can be further divided into two subpopulations according to the expression of Ly6C or Ly6G, namely mononuclear MDSCs and granulocyte MDSCs.^11^, ^12^ It has been observed that MDSCs accumulated in both peripheral blood and tumour tissues of cancer patients,^1^, ^13^ indicating their potential role in the tumour prognosis and development. Thus, MDSCs have received heat discussion.^14^, ^15^ MDSCs are known to have a dual role in tumour progression. While they control anti‐tumour immune responses, they also promote tumour angiogenesis, tumour cell invasion and pre‐metastatic niche formation.^16^, ^17^ The levels of MDSCs have a significant impact on clinical outcomes and therapeutic effects in patients with LC.^18^ LCs are known to exhibit high levels of MDSCs, which have been linked to resistance to chemotherapy, similar to other cancers.^1^ According to previous research, tumour‐derived factors such as growth factors and inflammatory cytokines or chemokines can recruit MDSCs from the bone marrow to peripheral blood,^19^ which profoundly influence the mobilization and activation of MDSCs.^20^ Some studies also describe tumour‐associated macrophages (TAMs) and tumour‐associated neutrophils (TANs) as descendants of MDSCs.^21^ Hence, MDSCs in mice are identified as immature bone marrow‐derived CD11b^+^Gr‐1^+^ cells composed of Ly6C^high^ monocytes and Ly6G^high^ granulocytes. In conclusion, the main paradigm is that TAM and TAN populations are derived from monocyte and granulocyte progenitors arising in the bone marrow.^21^ Indeed, it has been proved that circulating Ly6C^high^ monocytes accumulate within tumours and replenish the non‐proliferating TAM population,^22^ which in turn promotes tumour proliferation and invasion.^23^, ^24^ Thus, the immune function and cytotoxic effect of Ly6C^high^ monocytes migrating to tumour tissues are blocked. TANs can be recruited into the TME and function through chemokines secreted by tumour cells or immune cells during tumorigenesis.^21^, ^25^ Among the many chemokines or cytokines, MCP‐1 is a vital monocyte‐attracting chemokine with strong chemotaxis activity, and potently recruits blood monocytes to inflammatory or tumour sites^26^, ^27^; LTB4 is a chemokine with a strong effect on neutrophils, playing an important role in the chemotaxis and infiltration of neutrophils.^28^ Previous study pointed that suppressing lung cancer cell growth in mice is dependent on circulating macrophages and neutrophils.^29^ Macrophages are one of the primary cell types that drive the production of MCP‐1 and LTB4.^26^, ^30^

A previous study showed a parallel increase of Galectin‐3 level and the number of MDSCs in mice after cisplatin treatment,^31^ which provokes our interest. However, the relevant molecular mechanism of MDSC accumulation after cisplatin therapy is not well understood. Oxidative stress has been identified to be one of the primary contributors to the side effects of cisplatin.^5^ In cells, cisplatin forms a complex with the important mitochondrial antioxidant glutathione (GSH), consequently reducing mitochondrial GSH facilitated by organic cation transporter 1 (OCT1) and copper transporter 1 (Ctr1).^32^, ^33^ In addition, cisplatin induces dysfunction of electron transport chain I‐IV complexes, triggering the overproduction of reactive oxygen species (ROS).^34^ Oxidized phospholipid (oxPAPC) is a complex mixture of phospholipids that occur during physiological or pathological processes,^35^, ^36^, ^37^ which can be produced by two pathways: enzyme‐dependent oxidation or non‐enzyme‐dependent oxidation of phospholipids. Among them, ROS plays a vital role in the non‐enzyme‐dependent oxidation of phospholipids.^38^, ^39^, ^40^, ^41^, ^42^ In recent years, substantial evidence points that oxPAPC acts in the occurrence and development of inflammation and promote the occurrence and development of various acute and chronic inflammatory diseases.^43^, ^44^, ^45^ Indeed, some articles have pointed out that oxPAPC may also be inextricably linked to tumours, and they believe that platelet activating factor (PAF)‐like phospholipids can play a role in tumours by inducing systemic immunosuppression.^46^, ^47^, ^48^, ^49^ It is worth noting that oxPAPC was regarded as inducer of lung injury and cytokine production of lung macrophages.^50^ Thus, the critical questions regarding the possible role of oxPAPC in modulating inflammatory cells as well as the mechanisms in the TME remain to be answered.

In this work, we found an increased infiltration of inflammatory cells (including Ly6C^high^ monocytes and neutrophils) as well as oxPAPC level in tumour tissues after chemotherapeutic drugs treatment. Thus, we speculated that the inflammatory infiltration may be related to the overproduction of oxPAPC. Based on this hypothesis, we further explored the role of oxPAPC in inflammatory cells infiltration into LL2 lung tumour tissues after cisplatin treatment and its specific molecular pathways, aiming to provide ideas for clinically improvement of cisplatin on treating lung cancer.

## 2 METHODS AND MATERIALS

### 2.1 Cell culture

LL2, CT26 and MCF‐7 cells were purchased from the American Type Culture Collection. LL2 and MCF‐7 were cultured with folate‐free DMEM (Gibico) containing amikacin and 10% foetal bovine serum (FBS, Gibico) in incubators at 37°C and 5% CO_2_. CT26 cells were grown in RMPI‐1640 media (Gibico) containing amikacin and 10% FBS at 37°C, 5% CO_2_.

### 2.2 Animals

Specific‐pathogen‐free (SPF) female C57BL/6, BALB/c wild‐type mice and nude mice aged 6–8 weeks were purchased from HFK Bioscience (Beijing, China). SPF female CCL2−/− and LTB4R−/− mice were purchased from Jackson Laboratory (America), which were of a C57BL/6J background. The mice were housed in a 12‐h light/12‐h dark condition. This study was approved by the Institutional Animal Care and Use Committee of Sichuan University (Chengdu, Sichuan, China).

### 2.3 ROS measurement

Intracellular ROS was measured using cell‐permeable fluorescent 2′, 7′‐dichlorofluorescin diacetates (H2DCF‐DA) (Sigma). Cisplatin (10 μM), oxaliplatin (50 μM) and doxorubicin (10 μM) were used to treat LL2, CT26 and MCF‐7 cells (1 × 10^5^ cells/well) for 24 hours, respectively. Cells were harvested, washed with PBS, stained with 10 μM H2DCF‐DA for 30 min at 37°C, and analysed on a NovoCyte Flow Cytometer (ACEA Biosciences, Inc., San Diego, CA, USA). Fresh tumour tissues were collected, and immediately embedded in OCT compound following being sliced to a thickness of 6 μM. The sections were incubated with H2DCF‐DA (10 μM) at 37°C for 30 min and digitally photographed under a fluorescence microscope (Nikon Microsystems, Japan).

### 2.4 Cell necrosis and apoptosis assay

LL2, CT26 and MCF‐7 cells were seeded in 6‐well plates and respectively treated with cisplatin (10 μM), oxaliplatin (50 μM) and doxorubicin (10 μM) for 24 h. Then, cells were washed with PBS, re‐suspended in 200 μL Binding buffer containing 5 μL of Annexin‐V‐FITC and 5 μL propidium iodide (PI) (BD Biosciences) and incubated for 10 min at room temperature and protected from light. Samples were not stored, but analysed immediately.

### 2.5 Isolation of peritoneal primary macrophages

6–8‐week‐old female mice were sacrificed and soaked in 75% ethanol. 10 mL of pre‐chilled sterile saline was aspirated with a 20 mL syringe and i.p. injected into mice. After gently massaging the abdomen of mice, the intraperitoneal lavage liquid is slowly withdrawn with the syringe. Centrifuge the collected intraperitoneal lavage solution and discard the supernatant. If the collected cells contain red blood cells, resuspend the cells with sterile RBC lysate and then wash the cells with pre‐chilled PBS. The cells were then plated in 6‐well plates and cultured in RPMI 1640 medium containing 10% FBS, penicillin and streptomycin at 37°C for 2 h. Adherent cells can be regarded as primary pelagic macrophages.

### 2.6 ELISA

The levels of MCP‐1 and LTB4 in macrophage supernatant were measured by using Mouse CCL2/JE/MCP‐1 Quantikine ELISA Kit (BD Biosciences) and LTB4 ELISA Kit (Cayman Chemical), according to manufacturer's instructions.

### 2.7 Transwell assay

Bone cavities from femurs and tibias were flushed with FBS‐free medium to collect bone marrow cells and then filtered through a 70 μm nylon mesh following red blood cells lysis. The cell suspension was plated gently on a Histopaque‐1077/1119 gradient solution (Sigma) and centrifuged at 700 g for 30 minutes. Then, aspirating monocytes and neutrophils from the responding layer and cultured in RPMI 1640 medium containing 10% FBS, penicillin and streptomycin. Supernatants of peritoneal primary macrophages were collected 4 h after being treated with oxPAPC, PGPC or POVPC (Avanti Polar Lipid). Isolated monocytes or neutrophils were plated in upper chamber of Transwell culture plate, and 1640 RPMI medium, macrophage supernatant (MS), MS with MCP‐1 neutralizing antibody (BD Biosciences)/LTB4R inhibitor U‐75302 (Cayman Chemical), oxPAPC/PGPC/POVPC‐treated MS, oxPAPC/PGPC/POVPC‐treated MS with MCP‐1 neutralizing antibody/LTB4R inhibitor U‐75302 were added to the lower chamber. After 4 h of incubation, the number of migrated monocytes and neutrophils was counted by flow cytometry.

### 2.8 Tumour challenge and treatment experiments

Female C57BL/6, BALB/c and nude mice were respectively used for LL2 tumour model, CT26 tumour model, MCF‐7 tumour model. On day 0, LL2, CT26, MCF‐7 cells were collected and re‐suspended in serum‐ and penicillin‐free culture medium. To establish subcutaneous tumour model, mice were injected s.c. in the right back flank with 2 × 10^5^ LL2 tumour cells or 1 × 10^7^ MCF‐7 tumour cells. For peritoneal tumour model, 5 × 10^5^ CT26 tumour cells were injected i.p. When the tumours were palpable, oxaliplatin (10 mg/kg), cisplatin (5 mg/kg) and oxPAPC (10 mg/kg) were administered by i.p. injection, and ADM (10 mg/kg) was administered by i.v. injection.

### 2.9 Flow cytometry

To obtain single‐cell suspensions, tumour tissues were digested by 1 mg/mL collagenase Type IV (Sigma‐Aldrich) in RPMI 1640 basic medium for 2 h at 37°C. The intraperitoneal lavage liquid or ascites were obtained and centrifuged to prepare a single‐cell suspension. The red blood cell lysis buffer was then added to the single‐cell suspension, and digested cells were washed for three times and re‐suspended by PBS. Cells were dispersed in PBS at 1 × 10^6^ cells/mL and stained with 1 μL fluorescence‐conjugated antibodies (Biolegend, 1:100) for 30 min in 100 μL PBS at 4°C. Percp‐Cy5.5‐labelled rat anti‐mouse CD45, APC‐labelled CD11b, BV421‐labelled Ly6C and PE‐labelled Ly6G were used. We defined monocytes as CD45^+^CD11b^+^Ly6C^high^Ly6G^−^ and neutrophils as CD45^+^CD11b^+^Ly6C^−^Ly6G^+^. Samples were detected on NovoCyte Flow Cytometer (ACEA Biosciences, Inc., San Diego, CA, USA) and data were analysed by NovoExpress software (1.3.0, ACEA Biosciences, Inc., San Diego, CA, USA, 2018).

### 2.10 Immunohistochemistry and H&E staining

Tumour tissues of mice were fixed in 4% paraformaldehyde for 6 h and subsequently embedded in paraffin. Four‐micrometre thick sections were stained with haematoxylin and eosin (H&E) (BeyotimeInstitute of Biotechnology, Shanghai, China) for histomorphometric analysis. Immunohistochemistry analyses of tumour microenvironment were done with staining with E06, anti‐mouse CD11b, anti‐mouse Ly6G Ab (Servicebio) using the labelled streptavidin‐biotin method.

### 2.11 Statistical analysis

All quantitative data have been analysed using GraphPad Prism 9.4.0 (GraphPad, San Diego, CA, USA). p‐Values were calculated using a two‐tailed unpaired Student's t‐test, one‐way ANOVA, or two‐way ANOVA. Data were represented as mean ± SEM. A statistical probability of p < 0.05 was considered significant.

## 3 RESULTS

### 3.1 Ly6C^high^ monocytes and neutrophils significantly infiltrated into tumour tissues after chemotherapy

**FIGURE 1:**
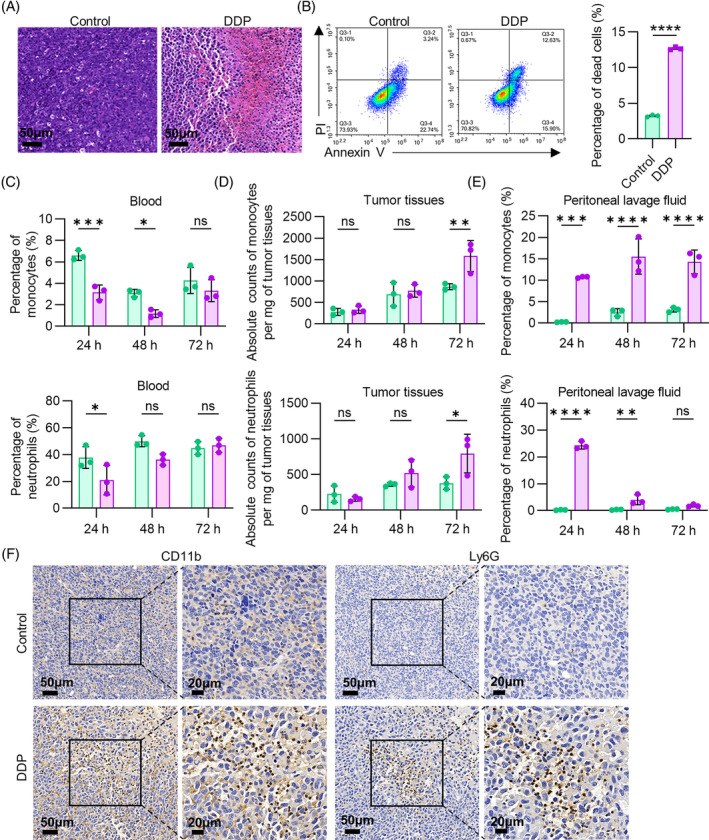
Ly6C^high^ monocytes and neutrophils significantly infiltrate into tumour tissues of LL2‐bearing mice after cisplatin treatment. (A) H&E staining of LL2 tumour tissues was performed 24 h after DDP treatment to observe the necrosis and inflammatory infiltration. (B) Flow cytometric analyses of PI‐Annexin V staining of necrotic LL2 cells treated with cisplatin (10 μM, 12 h). Annexin V^+^‐PI^+^ were considered necrotic cells. (C) Flow cytometric analyses of monocytes (CD45^+^CD11b^+^Ly6C^high^Ly6G^−^) and neutrophils (CD45^+^CD11b^+^Ly6G^+^Ly6C^−^) in peripheral blood post DDP treatment at different timepoint. (D) Flow cytometric analyses of monocytes and neutrophils in tumour microenvironment post DDP treatment at different timepoint. (E) Flow cytometric analyses of monocytes and neutrophils in peritoneal lavage fluid post DDP treatment. (F) IHC analysis of CD11b and Ly6G expression 72 h after DDP treatment. Data were shown as mean ± SEM, *n* = 3. *p < 0.05; **p < 0.01; ***p < 0.001; ns represents no significant difference.

Immune cells are critical components of the TME and have a significant impact on cancer progression. MDSCs are pathologically activated neutrophils and monocytes with remarkable immune suppressive activity. To explore whether chemotherapeutic drugs can increase infiltration of MDSCs into tumour tissues, we first established subcutaneous tumour model via subcutaneous injection of LL2 cells. Then, tumour‐bearing mice were administered with a standard therapeutic dose of cisplatin (DDP) (5 mg/kg body weight). 24 hours later, a lot of inflammatory cells appeared in the necrotic area of tumour tissues (Figure 1A). Prior to use, we have performed in vitro experiments where 10 μM cisplatin can cause extensive necrosis and apoptosis of LL2 cells, which confirms the effectiveness of cisplatin we used (Figure 1B). Monocytes and neutrophils were two types of abundant myeloid cells in TME. Thus, flow cytometry was performed to detect their proportion using established criteria: CD11b^+^Ly6C^high^Ly6G^−^ (M‐MDSC), CD11b^+^Ly6C^lo^Ly6G^+^ (PMN‐MDSC). The results showed that the proportion of Ly6C^high^ monocytes and neutrophils in peripheral blood were decreased at 24 h and 48 h time point (Figure 1C), while the significantly increased counts of monocytes and neutrophils in TME was detected 72 hours after cisplatin treatment (Figure 1D). The results of IHC also suggested that the expressions of CD11b and Ly6G were augmented after DDP treatment (Figure 1F). Besides, Ly6C^high^ monocytes and neutrophils in peritoneal lavage fluid were dramatically increased after DDP injection (Figure 1E). Thus, cisplatin treatment does lead to an increased migration of MDSCs to the TME. To investigate whether this phenomenon is universal, we also established CT26 peritoneal tumour model and MCF‐7 breast cancer model and treated with oxaliplatin and doxorubicin (ADM), respectively. Surprisingly, the necrosis sites of tumour tissues in both models were accompanied by extensive inflammatory cells infiltration (Figure S1A,B). In vitro, oxaliplatin and doxorubicin significantly caused necrosis of CT26 cells and MCF‐7 cells, respectively (Figure S1C). Similarly, flow cytometric analyses of the tumour tissues from these two tumour models demonstrated that the infiltration of Ly6C^high^ monocytes and neutrophils increased in TME to varying degrees (Figure S1D,E). MDSCs in peritoneal lavage fluid were dramatically increased after oxaliplatin treatment (Figure S1F). IHC analysis revealed that the expression of CD11b and Ly6G upregulated 48 hours after oxaliplatin and ADM treatment (Figure S1G,H). Collectively, it can be thought that chemotherapeutic agents can widely provoke the increased infiltration of MDSCs into TME.

### 3.2 Cisplatin induces ROS overproduction, and subsequently augments oxPAPC release to induce monocytes and neutrophils recruitment into tumour tissues

**FIGURE 2:**
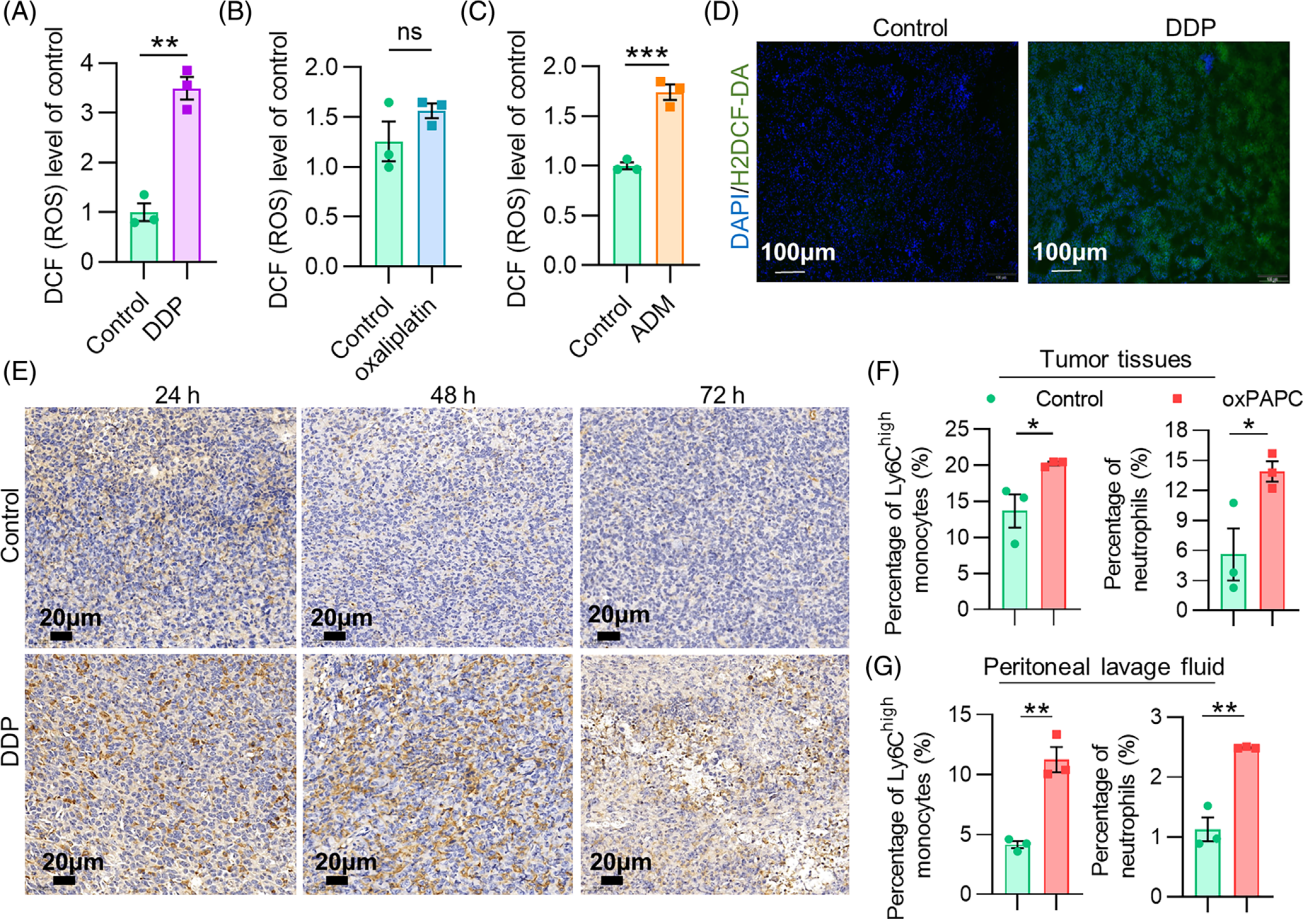
Cisplatin causes overproduction of ROS and oxPAPC, thus inducing monocytes and neutrophils recruitment into tumour tissues. (A–C) Flow cytometry was used to detect ROS levels in DDP/oxaliplatin/ADM‐respectively treated LL2/CT26/MCF‐7 cells after stained by H2DCF‐DA fluorescent probe. (D) ROS levels in LL2 tumour tissues from control and DDP‐treated mice were detected by H2DCF‐DA, DAPI‐stained nuclei are in blue. (E) Tumour tissues from control and DDP‐treated mice were stained with phospholipid‐specific antibody E06 at different time. (F) Flow cytometric analyses of monocytes and neutrophils infiltrated in the LL2 tumour tissues post oxPAPC treatment. (G) Flow cytometric analyses of monocytes and neutrophils in peritoneal lavage fluid of LL2‐bearing mice post oxPAPC treatment. Data were shown as mean ± SEM, *n* = 3. *p < 0.05; **p < 0.01; ***p < 0.001; ns represents no significant difference. The green bar represents the control group, the purple bar represents the DDP group, the blue bar represents the oxaliplatin group, the orange bar represents the ADM group and the red bar represents the oxPAPC‐treated group.

Cisplatin is a potent chemotherapy drug commonly used to treat many cancers, including lung cancer. However, it is associated with significant side effects due to its ability to produce ROS, causing oxidative stress and damage for normal cells. To examine the effect of cisplatin on the ROS production, we incubated cisplatin‐treated LL2 cells with an H2DCF‐DA fluorescent probe. Flow cytometric results suggested that ROS levels in LL2 cells began to increase dramatically 24 h after cisplatin treatment (1 μM, 24 h) (Figure 2A). Similar observations are appeared in CT26 and MCF‐7 cells, respectively treated with oxaliplatin and ADM (Figure 2B,C). Furthermore, we treated LL2‐bearing mice with cisplatin and sacrificed the mice 24 h later. Fresh tumour tissues were collected for frozen sections, performing dual immunostaining for DAPI and H2DCF‐DA fluorescent prob. Notably, the increased ROS in tumour tissues was more pronounced in the 24th hour after cisplatin treating (Figure 2D). Similar outcomes appeared in CT26 and MCF‐7 tumor models (Date not shown).

It is believed that ROS is one of the triggers for oxPAPC production. Accumulated studies have proved that oxPAPC is closely related to various inflammatory and immune diseases. Therefore, we assessed the oxPAPC level in tumour tissues after cisplatin treatment. Specifically, tumour tissues from control and cisplatin‐treated LL2 tumour‐bearing mice were stained with phospholipid‐specific antibody E06. After 24 hours, 48 hours and 72 hours of cisplatin treatment, the level of oxPAPC in cisplatin‐treated tumour tissues were substantially higher than that in control (Figure 2E), corroborating our initial hypothesis on cisplatin's potentiated increase of oxPAPC levels. Interestingly, we also observed that oxaliplatin and doxorubicin caused dramatically increases of ROS levels in vitro and in vivo (Data not shown). Meanwhile, there was a distinct trend in the increase of ROS and oxPAPC in tumour tissues from the MCF‐7 breast cancer model after ADM treatment than that from the CT26 celiac tumour model post oxaliplatin treatment (Data not shown). Thus, chemotherapy drugs may universally increase ROS levels in tumour tissues, triggering elevated oxPAPC release.

The induction of oxidative stress is a critical adverse effect associated with cisplatin treatment. ROS production is a hallmark of MDSC activity, which has been implicated in tumour progression and chemotherapy resistance. Previous studies suggest that oxPAPC is a crucial mediator of inflammation, and can be produced in response to oxidative stress. Considering these findings, we postulated that cisplatin‐induced oxidative stress may lead to the accumulation of oxPAPC in the TME, which might trigger the infiltration of MDSCs into tumour tissues. To verify this assumption, we established LL2 tumour model and examined the effects of oxPAPC in LL2 tumour model. After treating with oxPAPC, LL2‐bearing mice were sacrificed to be detected the infiltration of inflammatory cells in the tumour tissues and ascites. Flow cytometric results displayed increased recruitment of Ly6C^high^ monocytes and neutrophils in tumour tissues (Figure 2F), which was more remarkable in mouse intraperitoneal lavage fluid (Figure 2G). Together, these observations indicated that elevated ROS levels in tumour tissues caused by chemotherapeutic drugs can trigger the overproduction of oxPAPC, which may be one of the reasons that facilitate MDSCs infiltration into tumour.

### 3.3 MCP‐1 secreted by macrophages is responsible for the Ly6C^high^ monocytes chemotaxis caused by oxPAPC

**FIGURE 3:**
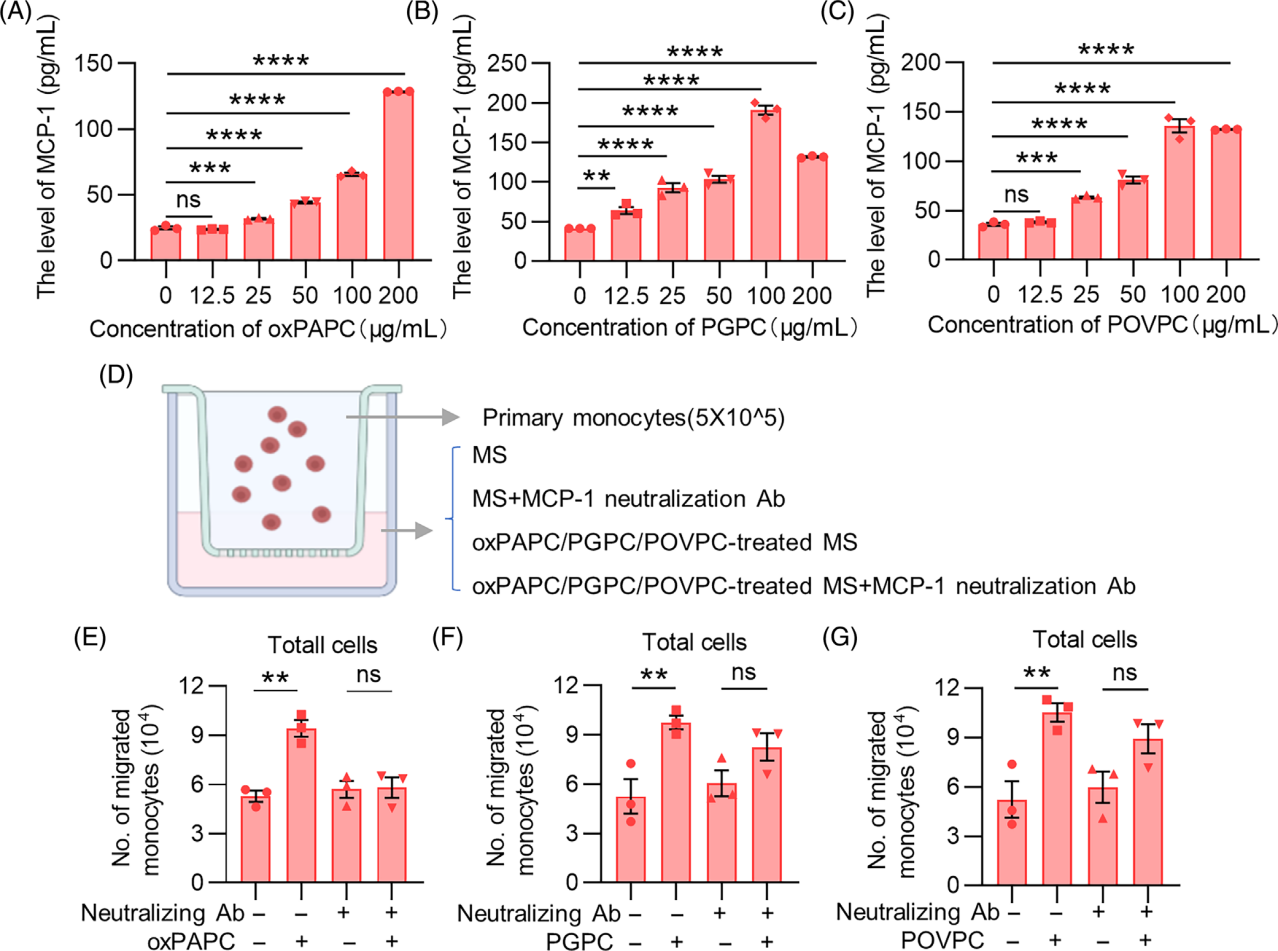
oxPAPC promotes monocytes chemotaxis in vitro via stimulating macrophages to secrete MCP‐1. (A–C) Extracted mouse primary macrophages were incubated with a series of concentration gradients of oxPAPC/PGPC/POVPC (0, 12.5 μg/mL, 25 μg/mL, 50 μg/mL, 100 μg/mL, 200 μg/mL) for 4 h. The content of MCP‐1 in culture supernatant was detected by ELISA. (D) 5 × 10^5^ primary monocytes were seeded into upper chamber (5 μm), and MS, MS with MCP‐1 neutralizing Ab (2.5 μg/mL), oxPAPC/PGPC/POVPC‐treated MS, oxPAPC/PGPC/POVPC‐treated MS with MCP‐1 neutralizing Ab (2.5 μg/mL) were added to the lower chamber. (E–G) After 4 h incubation, the average number of cells in the lower chamber were counted by flow cytometry. Data were shown as mean ± SEM, *n* = 3. *p < 0.05; **p < 0.01; ***p < 0.001; ns represents no significant difference.

Our studies above have proved that the infiltration of Ly6C^high^ monocytes and neutrophils in tumour tissues induced by cisplatin is relevant to oxPAPC. MCP‐1 can be secreted by macrophages, and can specifically stimulate monocytes chemotaxis, accelerating the development of cancer. Therefore, we hypothesized that oxPAPC induced the recruitment of Ly6C^high^ monocytes into tumour tissues through the mediation of MCP‐1. To demonstrate this, we stimulated the extracted mouse primary macrophages with a series of concentration gradients of oxPAPC (0, 12.5 μg/mL, 25 μg/mL, 50 μg/mL, 100 μg/mL, 200 μg/mL) for 4 h and the cell culture supernatant was collected to determine the content of MCP‐1. Surprisingly, when the oxPAPC concentration increased to 25 μg/mL, the content of MCP‐1 in supernatant began to increase compared to the control group, and then continued to upregulate in an oxPAPC dose‐dependent manner (Figure 3A). In fact, oxPAPC is a mixture of various oxidation products of PAP, mainly containing PGPC, POVPC, etc., and studies have shown that PGPC and POVPC play a different or even opposite role in many diseases. To gain further insight into the specific role of its main components in the macrophage secretion of MCP‐1, we treated mouse primary macrophages with the same concentration gradient of PGPC and POVPC, respectively, and then detected MCP‐1 level in the macrophage culture supernatant. When the concentration increased to 12.5 and 25 μg/mL, the content of MCP‐1 in the macrophage supernatant treated by PGPC and POVPC began to increase, respectively. The content of MCP‐1 reached the highest level when the concentration was 100 μg/mL, even higher than that at 200 μg/mL (Figure 3B,C). These results suggested that both the oxPAPC mixture and its main components can promote macrophage secretion of MCP‐1 to some extent.

To mimic the chemotaxis and recruitment of monocytes in mice after oxPAPC treatment, we performed a co‐culture system with Transwell membranes (5 μm). Isolated mouse primary monocytes were seeded in upper chamber, and MS (supernatant of macrophages), MS with MCP‐1 neutralizing antibody, oxPAPC‐treated MS, oxPAPC‐treated MS with MCP‐1 neutralizing antibody were added to the lower chamber (Figure 3D). After 4 h incubation, the average number of the total cells in the lower chamber were elevated by adding oxPAPC‐treated MS, and MCP‐1 neutralizing antibody effectively blocked oxPAPC‐induced chemotaxis of monocytes (Figure 3E). To further determine the specific effect of its main components on monocytes, we used PGPC and POVPC to replace oxPAPC to perform Transwell experiments in the same way (Figure 3D). The results showed that the treatment of PGPC and POVPC can improve the chemotactic effect of MS on monocytes, which can be inhibited by MCP‐1 neutralizing antibody (Figure 3F,G). Thus, the chemotactic effect on Ly6C^high^ monocytes after oxPAPC mixture or its main components treatment can be mediated by MCP‐1.

### 3.4 oxPAPC stimulates the migration of neutrophils via LTB4 secreted by macrophages

**FIGURE 4:**
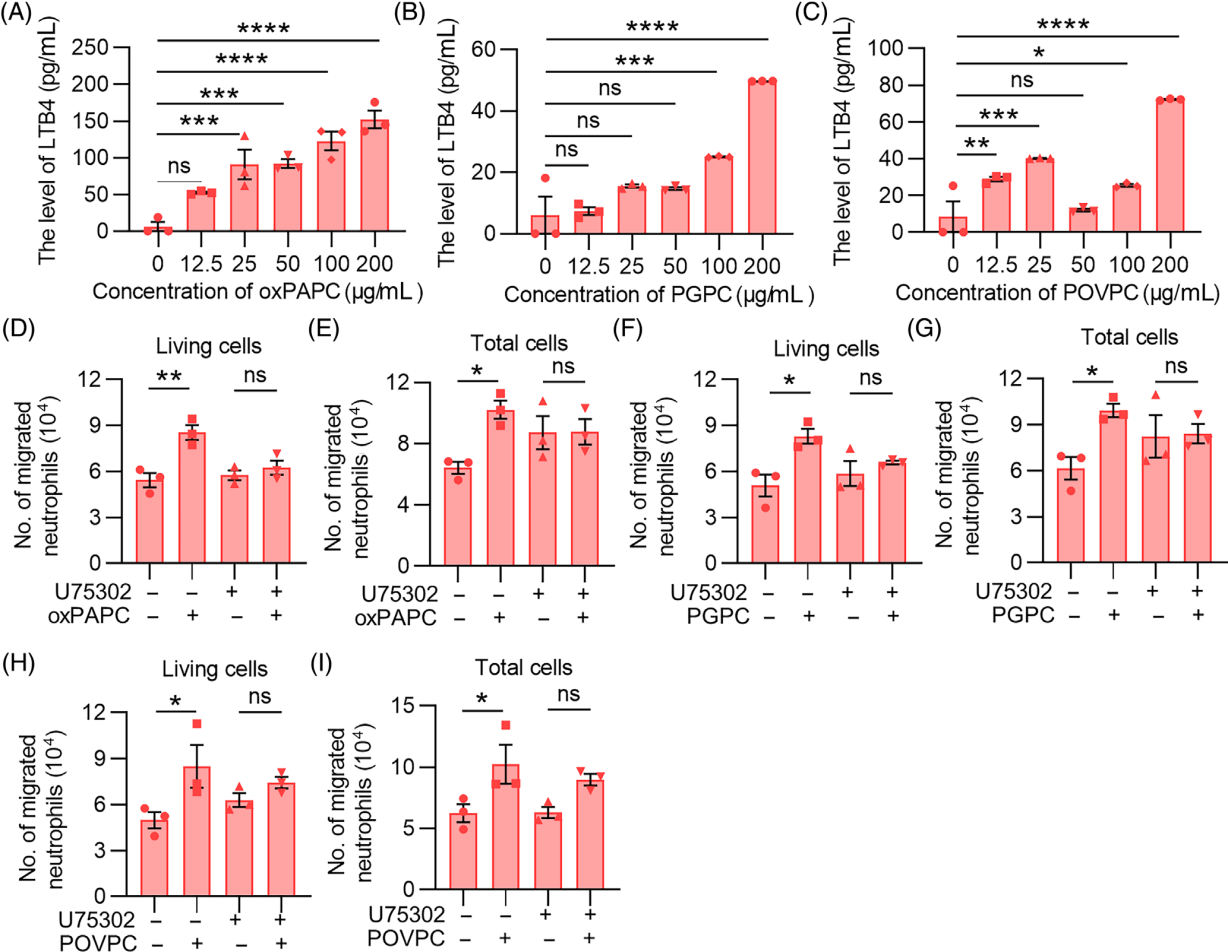
oxPAPC promotes neutrophils migration in vitro via stimulating macrophages to secrete LTB4. (A–C) Extracted mouse primary macrophages were incubated with a series of concentration gradients of oxPAPC/PGPC/POVPC (0, 12.5 μg/mL, 25 μg/mL, 50 μg/mL, 100 μg/mL, 200 μg/mL) for 4 h. The content of LTB4 in culture supernatant was detected by ELISA. (D–I) 5 × 10^5^ primary neutrophils were seeded into upper chamber (3 μm), and MS, MS with U75302, oxPAPC/PGPC/POVPC‐treated MS, oxPAPC/PGPC/POVPC‐treated MS with U75302 were added to the lower chamber. After 4 h incubation, the average number of viable cells and the total number of cells in the lower chamber were counted by flow cytometry. Data were shown as mean ± SEM, *n* = 3. *p < 0.05; **p < 0.01; ***p < 0.001; ns represents no significant difference.

Leukotriene B4 (LTB4) is a member of the leukotriene family, which is a powerful neutrophil chemokine that can be secreted by activated macrophages, acting in a variety of immune‐related diseases. In addition to trigger chemotaxis of Ly6C^high^ monocytes, oxPAPC has been shown to promote the infiltration of neutrophils into tumour tissues, indicating the cytokine‐mediated signalling network's complex nature in the TME. Hence, we tested the effect of LTB4 on neutrophil migration into tumour tissues by stimulating extracted primary macrophages from the abdominal cavity of mice with oxPAPC, PGPC and POVPC. The results showed marked upregulation of LTB4 in MS after exposing to oxPAPC mixture or its main components (Figure 4A–C), suggesting oxPAPC stimulates MS to secrete LTB4.

Additionally, similar chemotactic experiments were performed with primary neutrophils from mice. LTB4 receptor inhibitor U75302 was used to determine whether the migration of neutrophils is mediated through the LTB4/LTB4R pathway. Specifically, a co‐culture system with Transwell membrane (3 μm) was established. Isolated mouse primary neutrophils were seeded in upper chamber, and MS, MS with U75302, oxPAPC/PGPC/POVPC‐treated MS, oxPAPC/PGPC/POVPC‐treated MS with U75302 were added to the lower chamber. After 4 h incubation, the average number of viable cells and the total number of cells in the lower chamber were elevated by adding oxPAPC/PGPC/POVPC‐treated MS, and LTB4 receptor inhibitor U75302 effectively inhibited oxPAPC/PGPC/POVPC‐induced the secretion of LTB4 (Figure 4D–I). Collectively, these proved that the MS treated with oxPAPC/PGPC/POVPC has a chemotactic effect on neutrophils, and increases their migration mainly via LBL4/LTB4R axis.

### 3.5 oxPAPC treatment in vivo induced elevated MDSCs infiltration into tumour tissues through the MCP‐1/CCL2 and LTB4/LTB4R axis, which promoted tumour growth

**FIGURE 5:**
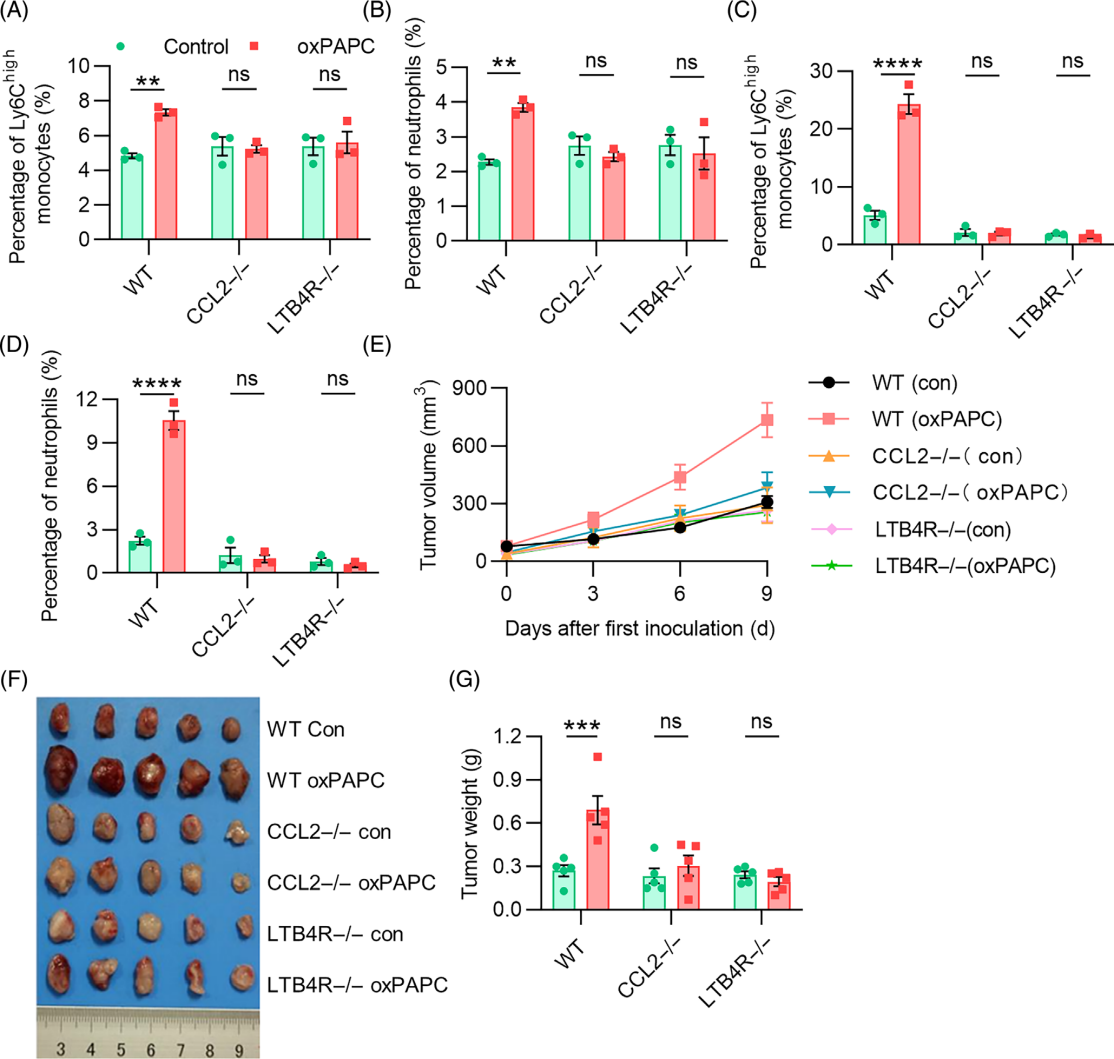
oxPAPC increases MDSCs infiltration into LL2 tumour tissues and thus promotes tumour growth through the MCP‐1/CCL2 and LTB4/LTB4R pathways. (A and B) Flow cytometric analyses of monocytes and neutrophils in tumour tissues from oxPAPC‐treated WT, CCL2−/− or LTB4R−/− mice. (C and D) Flow cytometric analyses of monocytes and neutrophils in ascites of oxPAPC‐treated WT, CCL2−/− or LTB4R−/− mice. (E) Tumour volume curve in each group. (F) Representative images of tumour nodules in each group. (G) Statistics of tumour nodules weight in each group. Data were shown as mean ± SEM, *n* = 3 or 5. *p < 0.05; **p < 0.01; ***p < 0.001; ns represents no significant difference.

The accumulation of MDSCs in the TME is associated with a high‐grade tumour, poor prognosis, and therapy resistance. Multiple studies suggest that various cytokines, including chemokines such as CCL2 and LTB4, are key factors that drive MDSC infiltration into the tumour microenvironment and promote their immunosuppressive functions. CCL2, also known as MCP‐1, is a potent recruiter of MDSCs and stimulates their migration to tumour sites. Similarly, LTB4 is a potent neutrophil and MDSC chemotactic factor that promotes their migration into the tumour microenvironment. Therefore, given the upregulated secretion of CCL2 and LTB4 in response to oxPAPC treatment, we aimed to investigate the possible mechanisms through which these cytokines promote the infiltration of MDSCs into tumour tissues. We established LL2 tumour‐bearing models in WT, CCL2−/− and LTB4R−/− mice, and treated them with oxPAPC. Tumour tissues were obtained for flow cytometric and immunohistochemical experiments 24 hours later. As we expected, the flow cytometric results showed that Ly6C^high^ monocytes and neutrophils markedly infiltrated in tumour tissues of oxPAPC‐treated WT mice, while there was no significant change in oxPAPC‐treated CCL2−/− mouse or LTB4R−/− mice compared with that in their respective control mice (Figure 5A,B). The percentage of Ly6C^high^ monocytes and neutrophils in ascites of oxPAPC‐treated WT, CCL2−/− or LTB4R−/− mice also perform the same tendency (Figure 5C,D). Immunohistochemical analysis of CD11b and Ly6G expression in tumour tissues further supported the results (Data not shown). Our findings conclusively showed that the increased infiltration of Ly6C^high^ monocytes and neutrophils into tumour tissues elicited by oxPAPC treatment in vivo is mediated by MCP‐1/CCL2 and LTB4/LTB4R signalling pathways.

Monocytes can produce anti‐tumor effectors to activate antigen‐presenting cells. On the other hand, monocytes can also bridge innate and adaptive immune responses, while conductive immune resistance, angiogenesis, and increased dissemination of tumour cells. Neutrophils can also be polarized into different activation states, triggering a variety of pro‐tumour or anti‐tumour functions. Our previous research has established the role of oxPAPC in promoting the infiltration of monocytes and neutrophils into the tumour microenvironment. However, it remains unclear whether this increase in inflammatory infiltration has a pro‐tumour or anti‐tumour effect. To address this critical question, we constructed LL2 subcutaneous tumour models of WT, CCL2−/−, and LTB4R−/− mice and treated them with oxPAPC on day 0 after tumour inoculation (10 mg/kg, intraperitoneal injection, once every other day). The tumour volume was measured every 3 days (Figure 5E). After 2 weeks, the mice were sacrificed and the tumour tissues were removed for weighing and photographing (Figure 5F,G). For WT mice, the tumour tissue volume growth rate and weight were greater after oxPAPC treatment. But for CCL2−/− and LTB4R−/− mice, there were not significantly distinct with their control group (Figure 5H–J). These findings clearly indicate that oxPAPC treatment in vivo do have a pro‐tumour effect and induce an increased MDSCs infiltration into tumour tissues through the MCP‐1/CCL2 and LTB4/LTB4R pathways.

### 3.6 oxPAPC contributes to cisplatin‐induced MDSCs recruitment into tumour tissues through the MCP‐1/CCL2 and LTB4/LTB4R pathways

**FIGURE 6:**
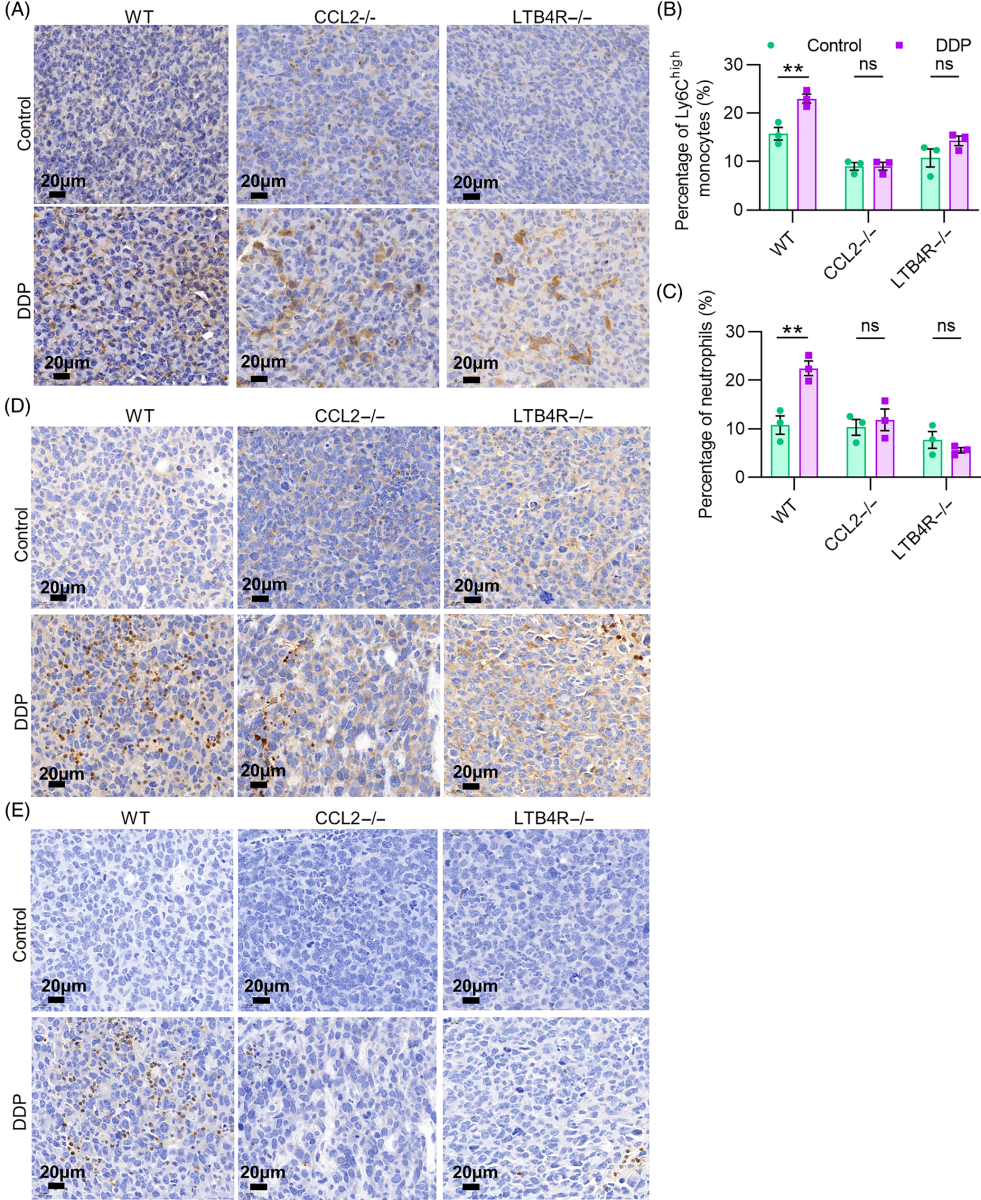
Cisplatin induces overproduction of oxPAPC, which promoted Ly6C^high^ monocytes and neutrophils infiltration in tumour through the MCP‐1/CCL2 and LTB4/LTB4R pathways. (A) Tumour tissues from control and DDP‐treated LL2 tumour‐bearing WT mice, CCL2−/− mice and LTB4R−/− mice were stained with phospholipid‐specific antibody E06. (B and C) The percentage of Ly6C^high^ monocytes and neutrophils in tumour tissues of DDP‐treated WT, CCL2−/− and LTB4R−/− mice. (D) IHC analysis of CD11b expression in tumour tissues of DDP‐treated WT, CCL2−/− or LTB4R−/− mice. (E) IHC analysis of Ly6G expression in tumour tissues of DDP‐treated WT, CCL2−/− or LTB4R−/− mice. Data were shown as mean ± SEM, *n* = 3. *p < 0.05; **p < 0.01; ***p < 0.001; ns represents no significant difference.

In order to better simulate the effect and mechanism of the Ly6C^high^ monocytes and neutrophils infiltration induced by cisplatin in vivo, we established LL2 tumour‐bearing model in WT mice, CCL2−/− mice and LTB4R−/− mice, and treated them with cisplatin. IHC results demonstrated that cisplatin caused upregulated expression of oxPAPC in all groups (Figure 6A). Further, we examined Ly6C^high^ monocyte and neutrophils infiltration in TME of each group. Similar to oxPAPC, cisplatin induced an increased infiltration of Ly6C^high^ monocytes and neutrophils in oxPAPC‐treated WT mice, while there was no remarkable increase in oxPAPC‐treated CCL2−/− mice or LTB4R−/− mice compared with that in their respective control mice (Figure 6B,C). Moreover, the expression of CD11b and Ly6G further confirmed it (Figure 6D,E). In summary, cisplatin provokes the widespread oxidation of phospholipids in tumour tissues and thus oxPAPC release to stimulate macrophages secreting MCP‐1 and LTB4, which recruit Ly6C^high^ monocytes and neutrophils to tumour tissues.

## 4 DISCUSSION

Within the global cancer burden, lung cancer is the leading cause of cancer‐related deaths. Platinum‐based chemotherapy is the first choice for lung cancer. Nevertheless, drug‐resistance and poor outcomes of patients limit the effect of platinum‐based drugs.^51^ TME plays a vital role in boosting and maintaining rapid tumour proliferation and differentiation, immune evasion, invasion and metastasis, as well as promoting the generation of tumour neovascularization.^52^, ^53^ Immunosuppressive cells, including MDSCs, TAMs and Treg cells, act as suppressive TME components to attenuate anti‐tumour immune responses.^54^ MDSCs have received increasing attention concerning their roles in lung cancer prognosis, development, and treatment.^14^, ^15^ In this work, we found the decreased percentages of inflammatory cells (including Ly6C^high^ monocytes and neutrophils) in peripheral blood and the augmented infiltrations of MDSCs in tumour tissues after chemotherapeutic drugs treatment. Thus, chemotherapeutic agents are likely to provoke an increased recruitment of immune cells into tumour tissues over a certain period of time, resulting in the augmented infiltration of MDSCs. Indeed, Ly6C^high^ monocytes are found in tumour tissues and induced to differentiate into TAMs, thereby promoting proliferation and invasion of tumour tissue.^55^, ^56^ N2‐type TANs can enhance the unlimited proliferation, invasion and metastasis of tumours, boost the generation of tumour neovascularization, and inhibit the function of anti‐tumour immune cells by activating TGF‐β‐mediated immunosuppression.^57^, ^58^ TAMs and TANs are regarded as descendants of MDSCs, and the increased infiltration of monocytes and neutrophils can likely promote tumour growth, thereby reducing the therapeutic effect of cisplatin. Nevertheless, the specific mechanism remains unclear.

Chemotherapeutic drugs have been shown to increase ROS levels in both tumour cells and tissues.^59^ Oxidative stress has been shown to be one of the primary contributors for the side effects caused by cisplatin.^5^ Cisplatin induces dysfunction of electron transport chain I‐IV complexes, triggering the overproduction of ROS.^34^ We successfully confirmed that the overproduction of ROS appeared in LL2, CT26 and MCF‐7 tumour models after chemotherapy. Oxidized phospholipid (oxPAPC) is a complex mixture of phospholipids that occur during physiological or pathological processes.^35^, ^36^, ^37^ ROS plays a pivotal role in the non‐enzyme‐dependent oxidation of phospholipids.^38^, ^39^, ^40^, ^41^, ^42^ Although a vast of studies have reported the role of oxPAPC in a variety of inflammatory diseases, few studies have mentioned the relationship between oxPAPC and tumours. We have conducted the study to investigate the role of oxPAPC in cancer and provide evidence to support its impact on tumour biology. Consistent with our findings on ROS, we also observed an increased release of oxPAPC in response to chemotherapy treatment. Most critically, oxPAPC enhanced the infiltration of monocytes and neutrophils into tumour tissues, promoting tumour growth in LL2 lung cancer model. Based on our research, we hypothesized that chemotherapeutic drugs caused the overproduction of ROS and ROS augmented the release of oxPAPC. In the following studies, we will delve into whether blocking ROS production would affect oxPAPC release, and what the exact mechanism of cisplatin‐ROS‐oxPAPC axis is.

oxPAPC can serve as damage‐associated molecular patterns (DAMPs) and mediate inflammatory activity in different ways.^60^ Among them, macrophages bind oxPAPC with its scavenger receptor CD36, and induce inflammatory activities via secreting cytokines.^60^ Based on current research, MCP‐1 and LTB‐4 can be secreted by macrophages and are potent inducers for monocytes and neutrophils, respectively. Consistent with this, our in vivo experiments proved that oxPAPC triggered macrophages secreting MCP‐1 and LTB‐4, which recruited monocytes and neutrophils into tumour respectively through the MCP‐1/CCL2 and LTB‐4/LTB4R axis. At last, we confirmed that cisplatin induces oxPAPC release, and thus promotes MDSCs infiltration to tumour via activating the MCP‐1/CCL2 and LTB4/LTB4R axis, potentially boosting tumour growth.

Generally speaking, a better understanding of how endogenous oxPAPC impact phagocyte function is crucial for uncovering the impact of oxPAPC on homeostasis and cancer. Despite our findings, several issues remain unresolved. It is not clear whether the primary source of oxPAPC is tumour cells or other cell types. In this regard, our knowledge is currently limited. Macrophages are not the only cell type contributing to the production of MCP‐1 and LTB4. Indeed, it is likely that there may exit other types of tumour stromal cells producing the cytokines, such as ECs, CAFs.^61^, ^62^, ^63^ We are supposed to track the origin of the MCP‐1 and LTB‐4, and further screen the chemokines that play the role to further verified our findings. Besides, we also did not figure out the relationship of Ly6C^high^ monocytes and TAMs, as well as neutrophils and TANs after cisplatin and oxPAPC treatment. Further research is needed in our follow‐up to study the infiltrated cells in tumour tissues with scRNA‐seq and spatial transcriptomics. While our research confirmed the pro‐tumour effects of oxPAPC in tumour‐bearing mice, there has been no studies regarding the possible correlation between the level of oxPAPC in the TME and response to chemotherapeutic drugs in cancer patients. In the future, we aim to conduct further research to address these issues.

## 5 CONCLUSION

**FIGURE 7:**
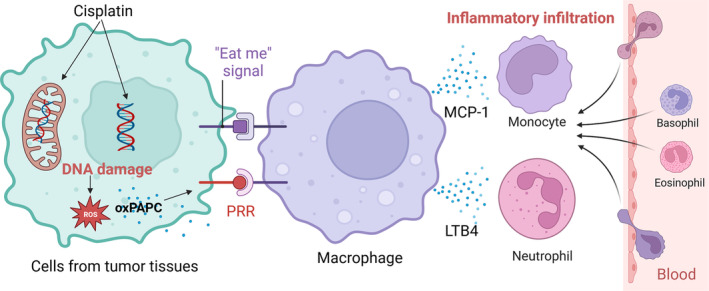
Mechanism diagram. Cisplatin induces oxPAPC release mediating MCP‐1/CCL2 and LTB4/LTB4R axis of macrophages to enhance inflammatory cell infiltration into tumour tissues.

To sum up, our results confirmed that cisplatin caused an upregulated infiltration of inflammatory cells (including Ly6C^high^ monocytes and neutrophils) in tumour tissues. Mechanically, cisplatin can induce an increased production of ROS in vitro and in vivo, which in turn provoked an upregulated oxPAPC release in tumour tissues. Further, oxPAPC triggered macrophages to secret MCP‐1 and LTB4 to promote chemotaxis and recruitment of monocytes and neutrophils to tumour tissues (Figure 7). We speculated that Ly6C^high^ monocytes gradually differentiated into TAMs, and neutrophils transformed into TANs after infiltrating into tumour tissues. Both TAMs and TANs play tumour‐promoting roles by promoting tumour proliferation and metastasis through multiple pathways, thereby reducing the therapeutic effect of cisplatin. Together, we uncover a potential mechanism for drug‐resistance produced by chemotherapeutic treatment, hoping to provide a novel strategy targeting oxPAPC to improve the traditional therapy against cancer.

## AUTHOR CONTRIBUTIONS

Ji Nie and Jiayuan Ai contributed equally to this work. *Study design*: Xiawei Wei and Qiu Sun. *Conducted the study*: Ji Nie, Jiayuan Ai, Weiqi Hong, Binhan Wang, Jingyun Yang, Ziqi Zhang, Fei Mo and Jing Yang. *Data collection and analysis*: Ji Nie, Jiayuan Ai, Weiqi Hong and Ziyi Bai. *Drafted and revised the manuscript*: Ji Nie and Jiayuan Ai. *Revised the final version of the manuscript*: Xiawei Wei and Qiu Sun. All authors take responsibility for the integrity of the data analysis.

## FUNDING INFORMATION

This work was supported by the National Science Foundation for Excellent Young Scholars (32122052) and National Natural Science Foundation Regional Innovation and Development (No. U19A2003).

## CONFLICT OF INTEREST STATEMENT

The authors declare no conflicts of interest.

## Supporting information


**Figure S1.** Oxaliplatin and doxorubicin induce MDSCs infiltration into CT26 and MCF‐7 tumour tissues, respectively. (A) H&E staining of CT26 tumour tissues 24 h after oxaliplatin treatment to observe the necrosis and inflammatory infiltration. (B) H&E staining of MCF‐7 tumour tissues 24 h after ADM treatment. (C) Flow cytometric analyses of necrotic CT26 cells treated with oxaliplatin (50 μM, 24 h) and necrotic MCF‐7 cells treated with ADM (10 μM, 24 h). (D) Flow cytometric analyses of monocytes and neutrophils infiltration in CT26 tumour tissues 48 h after oxaliplatin treatment. (E) Flow cytometric analyses of monocytes and neutrophils infiltration in MCF‐7 tumour tissues 48 h after ADM treatment. (F) Flow cytometric analyses of monocytes and neutrophils in peritoneal lavage fluid 48 h after oxaliplatin treatment. (G) IHC analysis of CD11b and Ly6G expression 48 h after oxaliplatin treatment. (H) IHC analysis of CD11b and Ly6G expression 48 hours after ADM treatment. Data were shown as mean ± SEM, *n* = 3. *p < 0.05; **p < 0.01; ***p < 0.001, ns represents no significant difference.

## Data Availability

The datasets in this study are available from the corresponding author on reasonable request.
