# Supplementary material for: Cisplatin‐induced oxPAPC release enhances MDSCs infiltration into LL2 tumour tissues through MCP‐1/CCL2 and LTB4/LTB4R pathways
Source: Cell Prolif. 2023 Oct 31;57(4):e13570. doi: 10.1111/cpr.13570 (PMC10984104; doi:10.1111/cpr.13570)
Supplement: Supplementary file 1 — Figure S1. Oxaliplatin and doxorubicin induce MDSCs infiltration into CT26 and MCF‐7 tumour tissues, respectively. (A) H&E staining of CT26 tumour tissues 24 h after oxaliplatin treatment to observe the necrosis and inflammatory infiltration. (B) H&E staining of MCF‐7 tumour tissues 24 h after ADM treatment. (C) Flow cytometric analyses of necrotic CT26 cells treated with oxaliplatin (50 μM, 24 h) and necrotic MCF‐7 cells treated with ADM (10 μM, 24 h). (D) Flow cytometric analyses of monocytes and neutrophils infiltration in CT26 tumour tissues 48 h after oxaliplatin treatment. (E) Flow cytometric analyses of monocytes and neutrophils infiltration in MCF‐7 tumour tissues 48 h after ADM treatment. (F) Flow cytometric analyses of monocytes and neutrophils in peritoneal lavage fluid 48 h after oxaliplatin treatment. (G) IHC analysis of CD11b and Ly6G expression 48 h after oxaliplatin treatment. (H) IHC analysis of CD11b and Ly6G expression 48 hours after ADM treatment. Data were shown as mean ± SEM, n = 3. *p < 0.05; **p < 0.01; ***p < 0.001, ns represents no significant difference. [file CPR-57-e13570-s001.docx]

**Supplement**

**
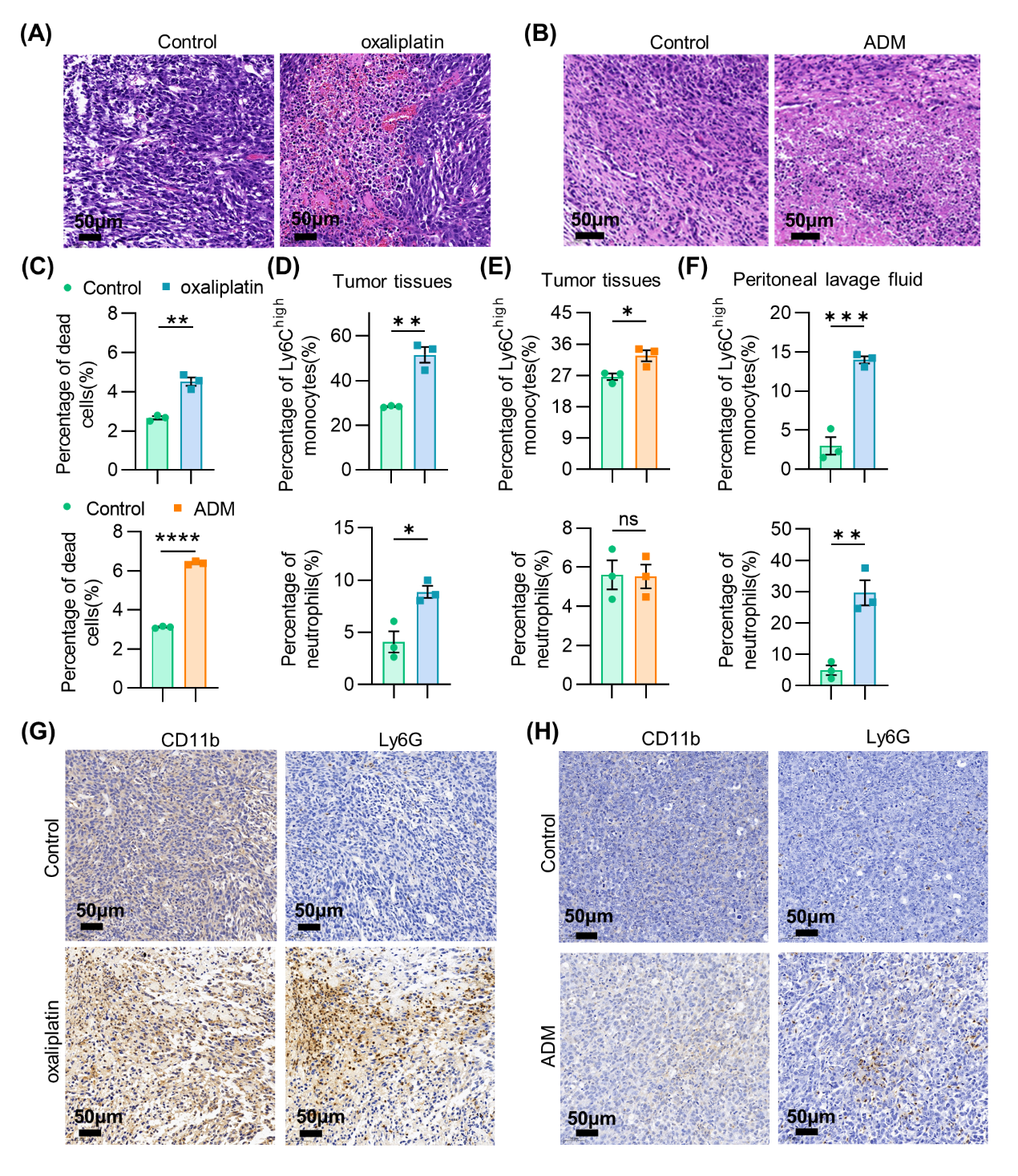
**

**Figure S1** Oxaliplatin and doxorubicin induce MDSCs infiltration into CT26 and MCF-7 tumor tissues, respectively**. (**A) H&E staining of CT26 tumor tissues 24h after oxaliplatin treatment to observe the necrosis and inflammatory infiltration. (B) H&E staining of MCF-7 tumor tissues 24h after ADM treatment. (C) Flow cytometric analyses of necrotic CT26 cells treated with oxaliplatin (50μM, 24h) and necrotic MCF-7 cells treated with ADM (10μM, 24h). (D) Flow cytometric analyses of monocytes and neutrophils infiltration in CT26 tumor tissues 48 hours after oxaliplatin treatment. (E) Flow cytometric analyses of monocytes and neutrophils infiltration in MCF-7 tumor tissues 48 hours after ADM treatment. (F) Flow cytometric analyses of monocytes and neutrophils in peritoneal lavage fluid 48 hours after oxaliplatin treatment. (G) IHC analysis of CD11b and Ly6G expression 48 hours after oxaliplatin treatment. (H) IHC analysis of CD11b and Ly6G expression 48 hours after ADM treatment. Data was shown as mean±SEM, n=3. *p <0.05, **p<0.01, ***p<0.001, ns represents no significant difference.
